# Supplementary material for: Cilastatin as a Potential Anti-Inflammatory and Neuroprotective Treatment in the Management of Glaucoma
Source: Int J Mol Sci. 2024 Mar 7;25(6):3115. doi: 10.3390/ijms25063115 (PMC10970106; doi:10.3390/ijms25063115)
Supplement: Supplementary file 1 [file ijms-25-03115-s001.zip › ijms-2843098-supplementary.pdf]

SUPPLEMENTARY MATERIAL

Table S1. Main significant results from the two-way ANOVA analysis of Brn3a expression in the different sub-regions of the retina 3 and 7 days after the OHT induction.

		Day 3			Day7		
		S[F=37.67; p<0.001]			S[F=11.68; p<0.001]<br>S*D[F=6.95; p=0.004]		
		Retinal regions			Retinal regions		
		Nasal	Central	Temporal	Nasal	Central	Temporal
Retinal zones	Superior	S[F=3.46; p=0.048]		S[F=14.99; p<0.001]<br>D[F=6.76; p=0.016]<br>S*D[F=3.86; p=0.035]		S*D[F=6.86; p=0.004]	S[F=7.7; p=0.003]<br>D[F=5.96; p=0.022]
	Central	S[F=7.02; p=0.004]	S[F=11.05; p<0.001]	S[F=10.76; p<0.001]	S[F=9.91; p<0.001]<br>S*D[F=8.91; p=0.001]	S[F=8.91; p=0.001]	
	Inferior	S[F=8.39; p=0.002]	S[F=9.53; p<0.001]	S[F=10.46; p<0.001]	S[F=4.87; p=0.017]<br>D[F=5.66; p=0.026]<br>S*D[F=4.04; p=0.031]		S[F=3.76; p=0.038]

Factors considered: surgical intervention (S), with three levels: eyes from mice not submitted to the surgery (NAÏVE), and from mice submitted to the laser-induced ocular hypertension surgery, the left-photocoagulated eye (OHT) and the contralateral one (CONTRA); and the pharmacological treatment (Drug, D), with two levels: cilastatin (CIL,300 mg/kg, i.p.) or vehicle (VH, saline) administered daily from two days before the surgery and until sacrifice. Degrees of freedom from the error: 24. In case of significant interactions post-hoc Tukey comparisons were performed. P-value, p. Brn3a: Brain-specific homeobox/POU domain protein 3A.

Table S2. Main significant results from the two-way ANOVA analysis of GFAP expression in the different sub-regions of the retina 3 and 7 days after the OHT induction.

		Day 3			Day7		
		S[F=6.67; p=0.005]<br>D[F=5.13; p=0.033]<br>S*D[F=3.6; p=0.043]			S[F=3.56; p=0.044]<br>D[F=4.86; p=0.041]		
		Retinal regions			Retinal regions		
		Nasal	Central	Temporal	Nasal	Central	Temporal
Retinal zones	Superior	D[F =6.12; p=0.021]		S[F =5.68; p=0.01]<br>S*D[F =6.15; p=0.007]	S[F =3.86; p=0.035]		S[F =3.59; p=0.043]
	Central		S[F =3.76; p=0.038]<br>D[F =6.17; p=0.02]<br>S*D[F =3.49; p0.047]	S[F =7.95; p=0.002]<br>S*D[F =10.73; p<0.001]			

**Inferior**		S[F = 6.67; p=0.005] S*D[F =5.9; p=0.008]	S[F =8.22; p=0.002] S*D[F =7.16; p=0.004]	D[F =5.34; p=0.03]	D[F =4.69; p=0.04]	S[F =3.92; p=0.034]

Factors considered: surgical intervention (S), with three levels: eyes from mice not submitted to the surgery (NAÏVE), and from mice submitted to the laser-induced ocular hypertension surgery, the left-photocoagulated eye (OHT) and the contralateral one (CONTRA); and the pharmacological treatment (Drug, D), with two levels: cilastatin (CIL,300 mg/kg, i.p.) or vehicle (VH, saline) administered daily from two days before the surgery and until sacrifice. Degrees of freedom from the error: 24. In case of significant interactions post-hoc Tukey comparisons were performed. P-value, p. GFAP: Glial fibrillary acidic protein.

Table S3. Main significant results from the two-way ANOVA analysis of GFAP expression in the different sub-regions of the inner and outer retina 3 and 7 days after the OHT induction.

		**Day 3**			**Day7**		
	**Inner**	S[F = 11.82; p<0.001] D[F = 7.76; p=0.01]			S[F = 4.63; p=0.02]		
	**Outer**						
		**Retinal regions**					
		**Nasal**	**Central**	**Temporal**	**Nasal**	**Central**	**Temporal**
**Retinal zones** **Superior**	**Inner**	S[F = 4.36; p=0.024] D[F = 7.78; p=0.01]	S[F = 6.51; p=0.006] S*D[F = 4.01; p=0.031]	S[F = 7.4; p=0.03] D[F = 6.19; p=0.02] S*D[F = 4.99; p=0.015]	S[F =4.17; p=0.028]		S[F =4.8; p=0.018]
	**Outer**			S*D[F = 3.97; p=0.032]			
**Central**	**Inner**	S[F =6.88; p=0.004]	S[F =5.15; p=0.014] D[F =5.74; p=0.025]	S[F =10.58; p=0.001] D[F =6.97; p=0.014] S*D[F =8.23; p=0.002]	D[F =4.97; p=0.035]	S[F =3.77; p=0.038] D[F =4.48; p=0.045]	
	**Outer**		S*D[F =5.04; p=0.015]	S*D[F =3.97; p=0.009]			D[F =5.2; p=0.032]
**Inferior**	**Inner**	S[F =7.99; p=0.002]	S[F =10.97; p<0.001]	S[F =10.08; p=0.001] D[F =6.21; p=0.02] S * D[F =5.8; p=0.009]	S[F =3.45; p=0.048] D[F =5.35; p=0.03]		S[F =4.19; p=0.027]

**Outer**	S*D[F =7.24; p=0.003]<br>No post-hoc differences		D[F =4.74; p=0.04]	D[F =5.05; p=0.034]	D[F =5.07; p=0.034]

Factors considered: surgical intervention (S), with three levels: eyes from mice not submitted to the surgery (NAÏVE), and from mice submitted to the laser-induced ocular hypertension surgery, the left-photocoagulated eye (OHT) and the contralateral one (CONTRA); and the pharmacological treatment (Drug, D), with two levels: cilastatin (CIL,300 mg/kg, i.p.) or vehicle (VH, saline) administered daily from two days before the surgery and until sacrifice. Degrees of freedom from the error: 24. In case of significant interactions post-hoc Tukey comparisons were performed. P-value, p. GFAP: Glial fibrillary acidic protein.

Table S4. Main significant results from the two-way ANOVA analysis of Iba-1 expression in the different sub-regions of the retina 3 and 7 days after the OHT induction.

		**Day 3**			**Day7**		
		S[F =229.67; p<0.001]<br>D[F =424.5; p<0.001]<br>S*D[F =141.37; p<0.001]			S[F =173.8; p<0.001]<br>D[F =96.84; p<0.001]<br>S*D[F =28.24; p<0.001]		
		**Retinal regions**					
		**Nasal**	**Central**	**Temporal**	**Nasal**	**Central**	**Temporal**
**Retinal zones**	**Superior**	S[F =21.31; p<0.001]<br>D[F =26.81; p<0.001]<br>S*D[F =11.55; p<0.001]	S[F =6.3; p=0.006]<br>D[F =21.44; p<0.001]<br>S*D[F =6.15; p=0.007]	S[F =5.16; p=0.014]<br>D[F =10.19; p=0.004]<br>S*D[F =6.26; p=0.007]	S[F =15.06; p<0.001]<br>D[F =6.71; p=0.016]	S[F =9.84; p<0.001]<br>D[F =14.92; p<0.001]<br>S*D[F =5.34; p=0.012]	S[F =17.65; p<0.001]<br>D[F =36.85; p<0.001]<br>S*D[F =5.1; p=0.014]
	**Central**	S[F =5.92; p=0.008]<br>D[F =12.87; p=0.001]	S[F =18.85; p<0.001]<br>D[F =34.85; p<0.001]<br>S*D[F =10.11; p<0.001]	S[F =45.33; p<0.001]<br>D[F =29.56; p<0.001]<br>S*D[F =20.04; p<0.001]	S[F =17.12; p<0.001]	S[F =67.6; p<0.001]<br>D[F =35.42; p<0.001]<br>S*D[F =19.2; p<0.001]	S[F =72.53; p<0.001]<br>D[F =38.74; p<0.001]<br>S*D[F =9.32; p=0.001]
	**Inferior**	S[F =17.46; p<0.001]<br>D[F =40.98; p<0.001]<br>S*D[F =3.49; p=0.047]	S[F =19.82; p<0.001]<br>D[F =36.43; p<0.001]<br>S*D[F =13.26; p<0.001]	S[F =21.78; p<0.001]<br>D[F =36.6; p<0.001]<br>S*D[F =19.3; p<0.001]	S[F =17.02; p<0.001]<br>D[F =11.43; p=0.002]	S[F =46.93; p<0.001]<br>D[F =28.67; p<0.001]<br>S*D[F =9.6; p<0.001]	S[F =28.25; p<0.001]<br>S*D[F =4.73; p=0.019]

Factors considered: surgical intervention (S), with three levels: eyes from mice not submitted to the surgery (NAÏVE), and from mice submitted to the laser-induced ocular hypertension surgery, the left-photocoagulated eye (OHT) and the contralateral one (CONTRA); and the pharmacological treatment (Drug, D), with two levels: cilastatin (CIL,300 mg/kg, i.p.) or vehicle (VH, saline) administered daily from two days before the surgery and until sacrifice. Degrees of freedom from the error: 24. In case of significant interactions post-hoc Tukey comparisons were performed. P-value, p. Iba-1: Ionized calcium-binding adapter molecule 1.

Table S5. Main significant results from the two-way ANOVA analysis of Iba-1 expression in the different layers of the retina 3 and 7 days after the OHT induction.

		**Day 3**	**Day 7**
**Retinal layers**	**ONFL-GCL**	S[F =21.31; p<0.001]<br>D[F =26.81; p<0.001]<br>S*D[F =11.55; p<0.001]	S[F =21.31; p<0.001]<br>D[F =26.81; p<0.001]<br>S*D[F =11.55; p<0.001]
	**IPL**	S[F =21.31; p<0.001]<br>D[F =26.81; p<0.001]<br>S*D[F =11.55; p<0.001]	S[F =21.31; p<0.001]<br>D[F =26.81; p<0.001]<br>S*D[F =11.55; p<0.001]
	INL	S[F =21.31; p<0.001]<br>D[F =26.81; p<0.001]<br>S*D[F =11.55; p<0.001]	S[F =21.31; p<0.001]<br>D[F =26.81; p<0.001]<br>S*D[F =11.55; p<0.001]
	**OPL**	S[F =21.31; p<0.001]<br>D[F =26.81; p<0.001]<br>S*D[F =11.55; p<0.001]	S[F =21.31; p<0.001]<br>D[F =26.81; p<0.001]<br>S*D[F =11.55; p<0.001]
	**ONL-PL**	S[F =21.31; p<0.001]<br>D[F =26.81; p<0.001]<br>S*D[F =11.55; p<0.001]	S[F =21.31; p<0.001]<br>D[F =26.81; p<0.001]<br>S*D[F =11.55; p<0.001]

Factors considered: surgical intervention (S), with three levels: eyes from mice not submitted to the surgery (NAÏVE), and from mice submitted to the laser-induced ocular hypertension surgery, the left-photocoagulated eye (OHT) and the contralateral one (CONTRA); and the pharmacological treatment (Drug, D), with two levels: cilastatin (CIL,300 mg/kg, i.p.) or vehicle (VH, saline) administered daily from two days before the surgery and until sacrifice. Degrees of freedom from the error: 24. In case of significant interactions post-hoc Tukey comparisons were performed. P-value, p. Iba-1: Ionized calcium-binding adapter molecule 1, ONFL-GCL: optic nerve fibers layer-ganglion cells layer, IPL: inner plexiform layer, INL: inner nuclear layer, OPL: outer plexiform layer, ONL-PL: outer nuclear layer-photoreceptor layer.
